# Supplementary material for: Zero-shot visual reasoning through probabilistic analogical mapping
Source: Nat Commun. 2023 Aug 24;14:5144. doi: 10.1038/s41467-023-40804-x (PMC10449798; doi:10.1038/s41467-023-40804-x)
Supplement: Supplementary file 1 — Supplementary Information [file 41467_2023_40804_MOESM1_ESM.pdf]

Supplementary Information for 'Zero-shot visual reasoning through probabilistic analogical mapping'

S1 Stimuli

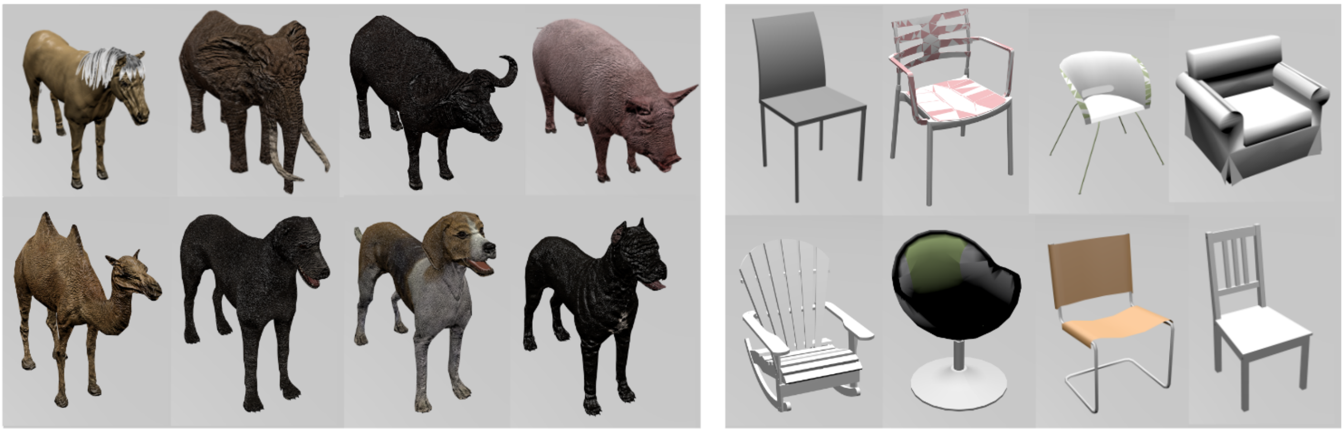

Figure S1: 3D Dataset. Eight chairs were selected from the ShapeNetPart dataset (each chair with a different shape), and eight animals from the Animal Pack dataset: horse, buffalo, domestic pig, camel, Hellenic hound, Celtic wolfhound, and Cane Corso.

S2 Error analysis

S2.1 2D image mapping

We conducted additional analyses to better understand visiPAM's strengths and limitations in the context of the 2D image mapping task. First, we performed a test in which the target image was horizontally reflected relative to the source image. This experiment allowed us to test the extent to which visiPAM's performance might depend on canonical orientation. We found that this manipulation did not affect visiPAM's performance for either within-category or between-category animal mapping (Table S1), suggesting that the model is robust to these kinds of low-level image manipulations.

	Within-category	Between-category
	Animals	
VisiPAM (default)	63.2% (59.1%)	67.9% (59.9%)
VisiPAM (target horizontally reflected)	62.7% (58.6%)	67.9% (59.9%)
Random	10%	20%

Table S1: VisiPAM's performance is robust to image manipulations. Mapping accuracy for both within-category (e.g., mapping from cat to cat, or horse to horse) and between-category (e.g., mapping from cat to horse) animal comparisons was largely unaffected by horizontal reflection of the target image. 'Random' denotes chance performance (determined by average number of part comparisons in each condition). Values in parentheses reflect chance-normalized performance (percentage of the range between chance performance and 100% accuracy).

We also evaluated the frequency with which visiPAM makes specific types of errors. Specifically, we computed the frequency with which each source part was mapped to each target part. The results of this analysis for both within-category and between-category animal comparisons are shown in Figures S2-S4. The most common type of error involved confusion of corresponding lateralized parts (e.g., confusion of left and right ears, or left and right legs). There were also occasionally errors related to spatial proximity of parts (e.g., confusion of the neck and head).

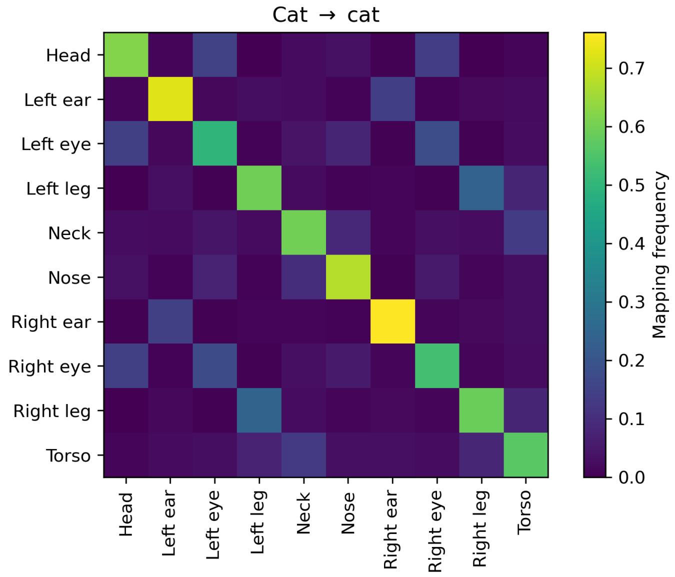

Figure S2: Error pattern for within-category animal mapping – cat to cat. Mapping frequency for each pair of source (rows) and target (column) parts. Perfect performance would correspond to a mapping frequency of 1 for all entries along the diagonal.

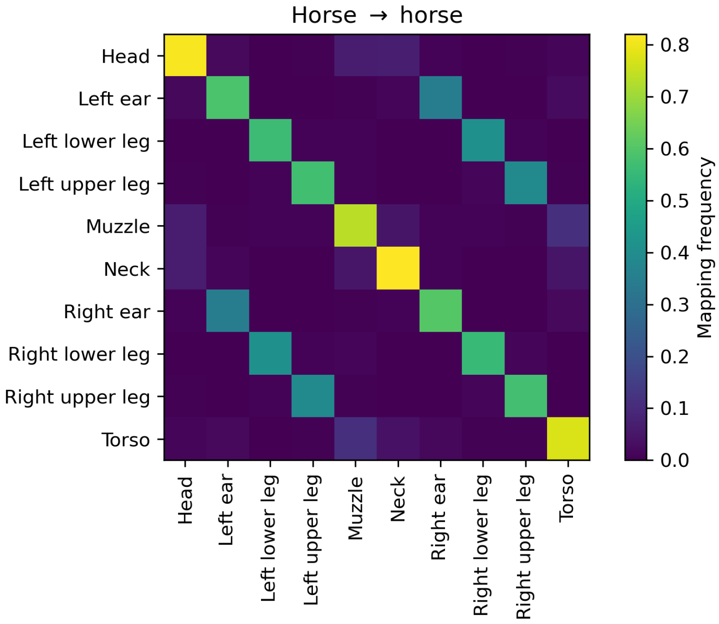

Figure S3: Error pattern for within-category animal mapping – horse to horse.

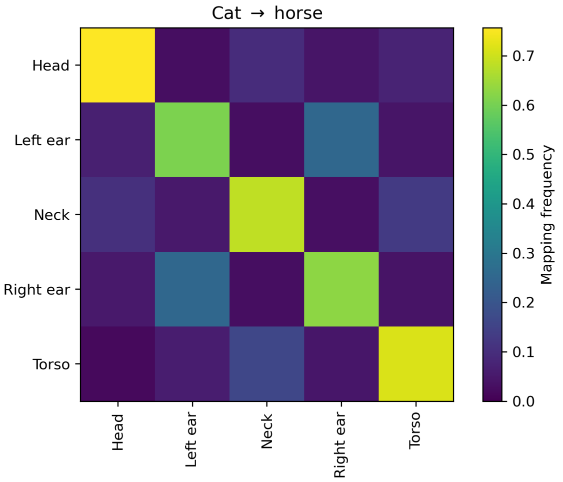

Figure S4: Error pattern for between-category animal mapping – cat to horse.

S2.2 3D object mapping

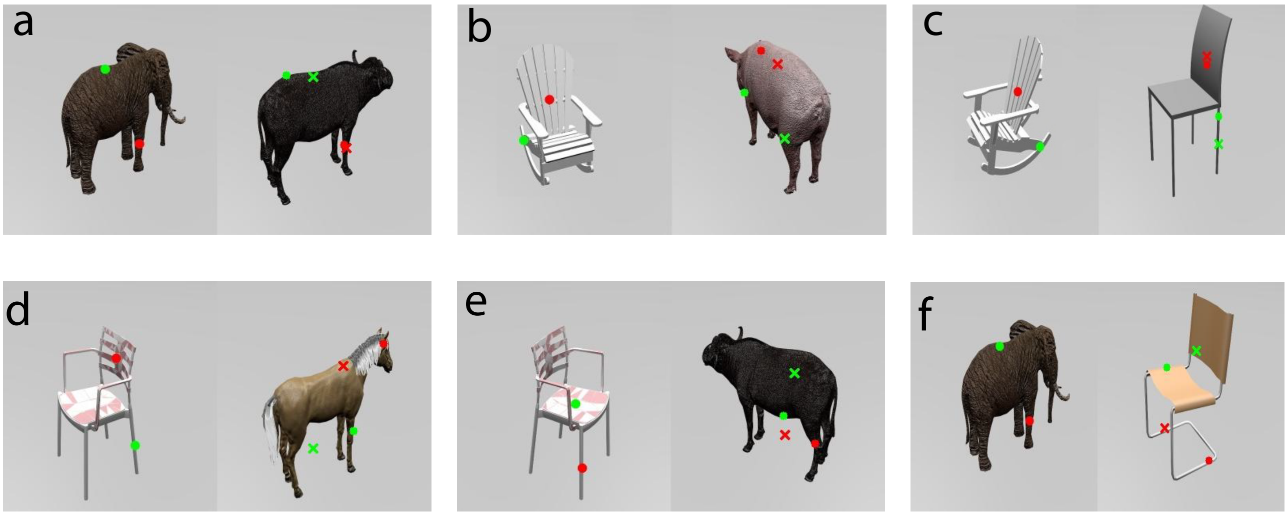

Figure S5: Examples of errors made by visiPAM on 3D mapping task. In each panel, images on the left panel are source images, and images on the right are target images that show both visiPAM predictions (circle) and human response centers (cross).

We conducted a qualitative analysis of the errors generated by visiPAM in the context of the 3D marker mapping experiment. We identified two primary causes of errors in predicting human marker placements: (1) the model's over-reliance on local 3D geometric properties (e.g., curvature, corner, T-junctions); and (2) a lack of functional knowledge or semantic information pertaining to the parts. Figure S5 illustrates a few examples in which visiPAM gives undue attention to local geometric structure in its mapping decisions. Circles indicate visiPAM's predicted mapping locations, and crosses indicate the mean location of human responses. For example, for the image pairs in Figure S5a, visiPAM maps the green marker on the back of the elephant (source) to a position (green circle) located on the lower back of the ox (target). For this mapping, the model largely relies on local geometric commonalities due to their similar surface curvature. Humans, on the other hand, map the green dot to the middle of the ox's spine based on knowledge of overall body structure. In Figures S5b and S5c, the model maps the green marker using local geometric cues of the corner (L junction). In contrast, humans use functional knowledge to select the back leg of the target object (green cross), which provides support for the object. The mapping of chairs in Figure S5d shows a similar mapping error (red circles) based on shared vertical surfaces, whereas humans appear to rely on semantic knowledge to match the "back" of the chair to the "back" of the horse. Similarly, in Figure S5e, the model maps the green marker on the basis of shared flat surfaces (e.g., seating surface of a chair, and flat belly of an ox), whereas humans are sensitive to the functional support relations. Lastly, Figure S5f shows an interesting case in which the model maps the front leg of the elephant (red marker) to a horizontal bar of the chair. In this instance, the model matches the back legs of the elephant to the chair's vertical legs; accordingly, because the model favors one-to-one mapping, the two front legs of the elephant are mapped to the remaining narrow surfaces (despite the mismatch of orientations).

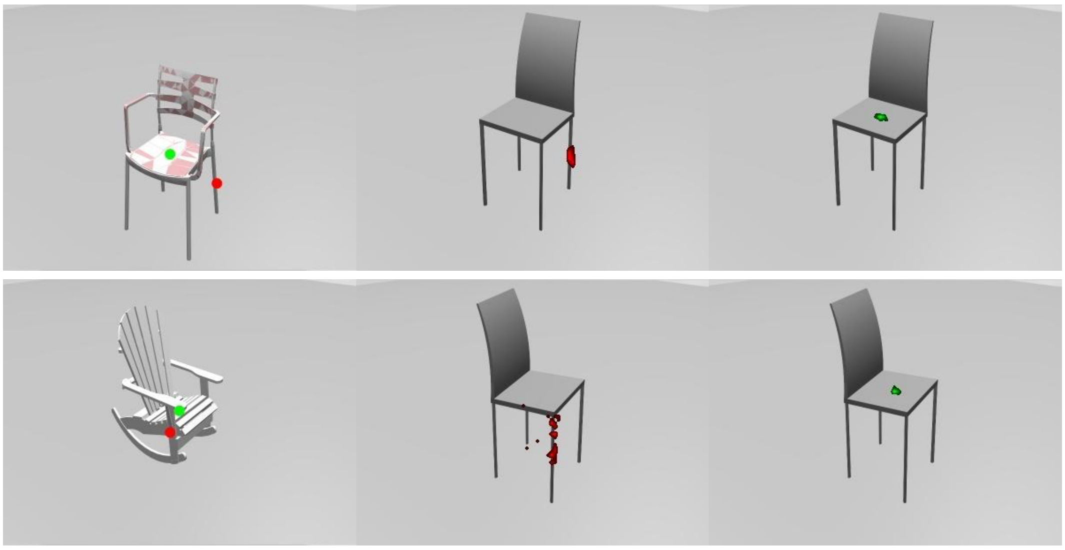

Figure S6: Explanation of increased variability in chair-to-chair condition. Both visiPAM and human participants displayed higher variability in the chair-to-chair condition than in the animal-to-animal condition. This difference was likely driven by the inherent diversity of chair shapes (see objects in Figure S1). To illustrate this, we have displayed two example problems. When the target and source chair had a similar shape (top panel), mappings had very low variability (heatmaps display distribution of human mapping judgments). When the target and source chair had very different shapes (bottom panel), mappings had much higher variability.

S3 Consistency analysis

A noteworthy phenomenon is the consistent utilization of semantic, functional and visual information in human judgments when deciding the relative relation between the two marker locations. As illustrated in the right panel of Figure 3b (main text), this tendency resulted in the formation of two distinct clusters for each marker in the between-category condition, with one cluster reflecting semantic knowledge (mapping the red dot on the back of the chair to the back of the horse) and the other based on spatial similarity (mapping the back of the chair to the neck

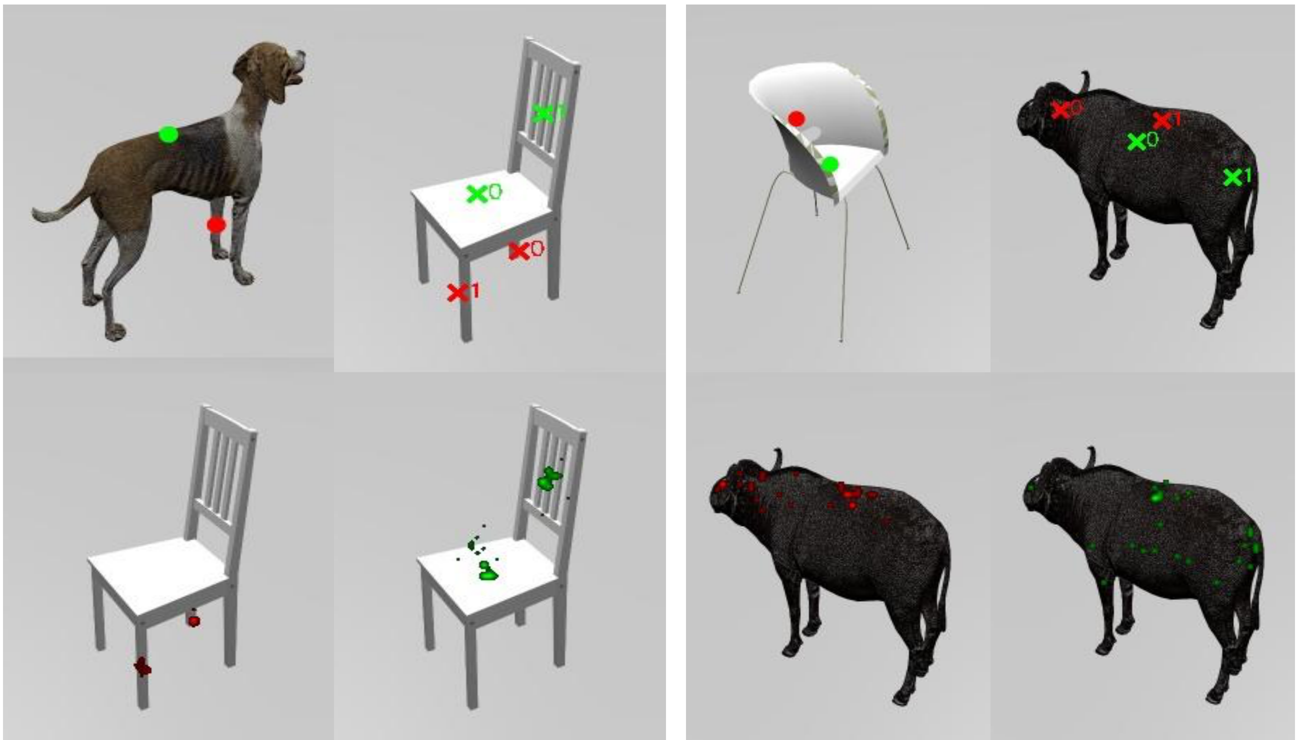

Figure S7: Examples illustrating marker consistency. Two example problems are shown. For each problem, human mapping judgments were bimodal, with two clusters of responses for each of the green and red markers. These clusters were generally consistent with a strategy that was based on either spatial relational information or semantic information. The clusters labeled with the index 1 follow a semantic strategy, e.g. mapping the back of the dog to the back of the chair (left panel), or mapping the back of the chair to the back of the buffalo (right panel). The clusters labeled with the index 0 follow a spatial strategy, e.g. mapping the back of the dog to the seat of the chair. Human responses were most often consistent, in the sense that both marker placements were governed either by a semantic strategy or a spatial strategy.

of the horse). Depending on the mapping chosen for the red dot (the back of the chair corresponds to the back or neck of the horse), the green dot was placed on different legs of the horse.

To systematically analyze whether humans make consistent judgments for the two marker placements, we conducted the following analysis. First, we employed a dip test (with a threshold of $p < 0.05$) to determine whether marker locations reported by human participants had a bimodal distribution. The dip test revealed 22 trials in which both red and green markers were bimodally distributed. For these trials, we used the KMeans++ algorithm [53] to segregate responses into two clusters. We proceeded to examine the consistency between the reported marker locations. In most of these 22 problems, we observed consistency in the type of information utilized by participants, with both markers being consistently matched based on either visual or semantic information.

Figure S7 presents two examples that illustrate marker consistency. Each marker in the figure has two cluster centers with the same color, and a number plotted next to the cluster center indexing the cluster. Out of 41 participants, 15 placed the green and red markers in cluster 0 in the left panel of the four images, whereas 16 placed the markers in cluster 1; 10 other participants did not follow a semantic or visual pattern. In this context, cluster 0 represents mapping primarily based on geometric properties in the object, indicating that participants relied on visual similarity between the source and target objects. In contrast, cluster 1 is a mapping based on semantic knowledge: mapping the back of the dog to the back of the chair despite their different local geometry properties. When participants mapped the back of the dog to the back of the chair, the front of the chair is on the left of the chair image. Hence, the front leg of the dog (red dot) would map to the cluster-1 location of the chair.

Mapping consistency was also observed in the mapping between a chair and a buffalo (right panel). Specifically, out of the total of 41 participants, 13 performed mapping based on visual similarity, placing both the green and red markers in cluster 0. In contrast, 24 participants relied on semantic similarity, placing the markers in cluster 1.

The other 4 participants did not show any apparent pattern in their judgments. These analyses revealed a general pattern of consistent mapping among the participants, with a majority utilizing semantic information for mapping. Overall, across the 22 between-category mapping problems (for which 41 participants yielded a total of 902 trials), 568 trials (63%) showed a consistent mapping strategy for human judgments.

An examination was also conducted to determine if visiPAM exhibited a consistent mapping strategy. The results revealed that in 19 out of the 22 problems, VisiPAM consistently applied the mapping strategy based on visual similarity. Thus, similar to human participants, visiPAM was generally consistent in the mappings that it produced.

S4 Cluster analysis for 3D object mapping

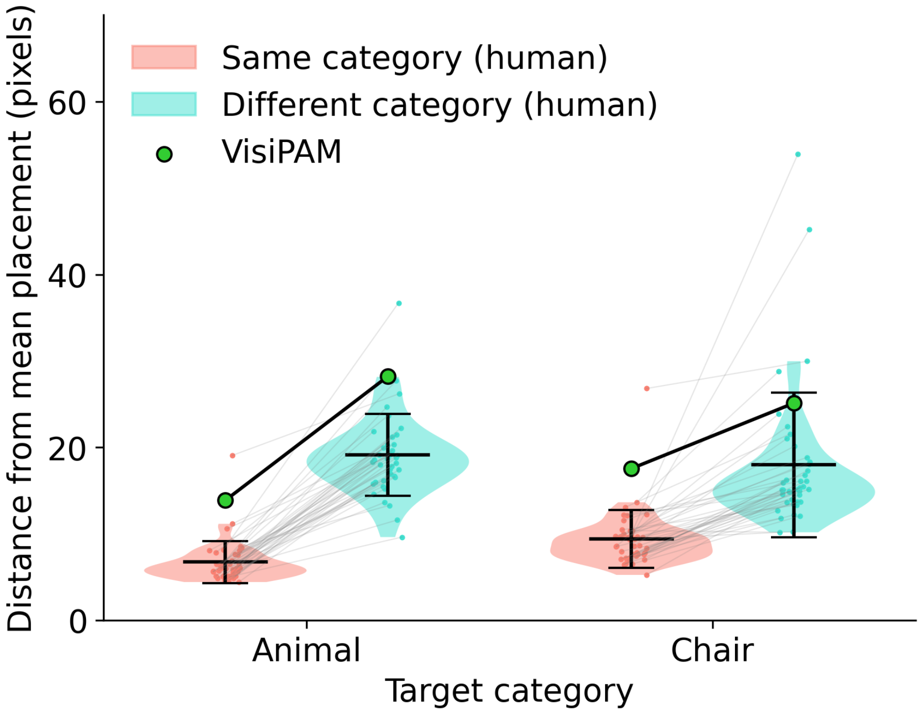

Figure S8: Comparison of visiPAM with human behavior when controlling for bimodal response distribution. Violin plot comparing human placements and visiPAM predictions. Each problem was classified as having either a unimodal or bimodal response distribution (for human responses) using a dip test (with a threshold of $p < 0.05$). For problems with a unimodal distribution, responses were quantified based on the distance to the overall mean for that trial (as in the primary results displayed in Figure 5). For problems with a bimodal distribution, a clustering analysis was performed to sort responses into two clusters, and responses were quantified based on the distance to the mean of the closest cluster. This was done for both visiPAM and human responses. All problems in the same-superordinate-category condition were classified as unimodal (thus the results in this condition are identical to Figure 5). The dip test classified 48.4% of problems in the different-superordinate-category condition as bimodal. Even after accounting for this bimodality, responses were still more variable in the different-superordinate-category condition (for both humans and visiPAM). Strip plots (small colored dots) indicate average distance in each condition, one point for each participant ($N = 41$). Thin gray lines show the within-participant differences for the same- vs. different-superordinate-category conditions. Violin plots exclude data points greater than 2.5 standard deviations away from mean, though these individual data points are included in strip plots. Black horizontal lines indicate mean human distances in each condition, and error bars indicate standard deviations. Large green dots indicate visiPAM predictions (i.e., the distance between model predicted location and human mean location).

S5 Ablation analyses

S5.1 2D image mapping

VisiPAM's edge embeddings involved two different types of spatial relations, one based on angular distance ($\mathbf{r}_\theta$), and one based on vector difference ($\mathbf{r}_\delta$; see Section 4.2.3 for more details). We performed ablation experiments in which visiPAM's edge embeddings only contained one of these spatial relation types. The results demonstrated that both components contributed significantly to visiPAM's performance (Table S2).

	Within-category		Between-category
	Animals	Vehicles	Animals
**VisiPAM**	**63.2% (59.1%)**	**69.5% (58.8%)**	**67.9% (59.9%)**
VisiPAM ($\mathbf{r}_\theta$ only)	56.7% (51.8%)	66.7% (55%)	67.5% (59.4%)
VisiPAM ($\mathbf{r}_\delta$ only)	56.1% (51.2%)	65.4% (53.2%)	52.5% (40.6%)
Random	10%	26%	20%

Table S2: Analogical mapping with 2D images – edge embedding ablation analyses. Mapping accuracy on part-matching task. VisiPAM performed worse when edge embeddings were based only on angular distance ($\mathbf{r}_\theta$) or vector difference ($\mathbf{r}_\delta$). Bold text indicates the best performing model. 'Random' denotes chance performance (determined by average number of part comparisons). Values in parentheses reflect chance-normalized performance (percentage of the range between chance performance and 100% accuracy).

We also carried out an additional experiment to determine whether visiPAM's performance can be further improved by incorporating additional sources of relational information. Specifically, we augmented visiPAM's edge embeddings with information about whether two parts are connected. This is an important aspect of relations between object parts that may not be entirely captured by simple spatial relations, especially for non-rigid body objects such as animals. For instance, the spatial relation between two body parts (e.g., the hand and the shoulder) can change dramatically depending on posture, but the topological relationship between body parts (e.g., which body parts are connected) remains unchanged. To test whether this information might further improve visiPAM's performance, we tested a model in which edges were modified based on whether the corresponding object parts were connected (using ground-truth knowledge). For parts that were not connected, the values for the spatial relation components ($\mathbf{r}_\theta$ and $\mathbf{r}_\delta$) were set to 0, while spatial relation embeddings were left unchanged for connected parts. Additionally, an extra dimension was added to the edge embeddings with a value of 1 for connected parts, and a value of -1 for non-connected parts. We found that this version of visiPAM performed even better than the version that included spatial relations only, particularly for mapping problems involving animals (Table S3). Though this preliminary experiment relied on ground-truth knowledge about the object parts, future work might explore methods for extracting this information directly from images.

Within-category		Between-category
Animals	Vehicles	Animals
72.4% (69.3%)	70.3% (59.9%)	77% (71.3%)

Table S3: VisiPAM's performance is improved by incorporating topological relations. VisiPAM's performance was improved when edges were augmented with information about whether two parts were connected. Values in parentheses reflect chance-normalized performance (percentage of the range between chance performance and 100% accuracy).

S5.2 3D object mapping

	Same superordinate category		Different superordinate category		
	Animal-to-animal	Chair-to chair	Animal-to-chair	Chair-to-animal	r
**VisiPAM**	**11.51**	**17.03**	**34.37**	**36.63**	**0.70**
VisiPAM (nodes only)	13.77	19.96	35.83	37.98	0.61
VisiPAM (edges only)	12.61	19.32	34.86	40.10	0.60

Table S4: Ablation results for comparison of visiPAM with human behavior. VisiPAM performed better than ablated models based on either node or edge similarity only (as indicated by bold text). Results in the middle four columns reflect average distance between visiPAM and human marker placements (smaller values indicate better fit to human responses) in each of four conditions, defined by the target category (animal vs. chair), and whether or not the source and target objects were from the same (e.g., animal-to-animal) or different (e.g., animal-to-chair) superordinate categories. Results in the rightmost column reflect the item-level correlation between human and visiPAM distances to the human mean placement (higher values indicate a better fit to human responses).
