# Supplementary material for: Zero-shot visual reasoning through probabilistic analogical mapping
Source: Nat Commun. 2023 Aug 24;14:5144. doi: 10.1038/s41467-023-40804-x (PMC10449798; doi:10.1038/s41467-023-40804-x)
Supplement: Supplementary file 3 — Reporting Summary [file 41467_2023_40804_MOESM3_ESM.pdf]

nature portfolio

Corresponding author(s): Taylor Webb

Last updated by author(s): Jul 3, 2023

Reporting Summary

Nature Portfolio wishes to improve the reproducibility of the work that we publish. This form provides structure for consistency and transparency in reporting. For further information on Nature Portfolio policies, see our Editorial Policies and the Editorial Policy Checklist.

Statistics

For all statistical analyses, confirm that the following items are present in the figure legend, table legend, main text, or Methods section.

n/a	Confirmed	
☐	☒	The exact sample size (*n*) for each experimental group/condition, given as a discrete number and unit of measurement
☐	☒	A statement on whether measurements were taken from distinct samples or whether the same sample was measured repeatedly
☐	☒	The statistical test(s) used AND whether they are one- or two-sided<br>*Only common tests should be described solely by name; describe more complex techniques in the Methods section.*
☐	☒	A description of all covariates tested
☐	☒	A description of any assumptions or corrections, such as tests of normality and adjustment for multiple comparisons
☐	☒	A full description of the statistical parameters including central tendency (e.g. means) or other basic estimates (e.g. regression coefficient) AND variation (e.g. standard deviation) or associated estimates of uncertainty (e.g. confidence intervals)
☐	☒	For null hypothesis testing, the test statistic (e.g. F, t, r) with confidence intervals, effect sizes, degrees of freedom and P value noted<br>*Give P values as exact values whenever suitable.*
☒	☐	For Bayesian analysis, information on the choice of priors and Markov chain Monte Carlo settings
☒	☐	For hierarchical and complex designs, identification of the appropriate level for tests and full reporting of outcomes
☒	☐	Estimates of effect sizes (e.g. Cohen's *d*, Pearson's *r*), indicating how they were calculated

Our web collection on statistics for biologists contains articles on many of the points above.

Software and code

Policy information about availability of computer code

Data collection	All model simulations were carried out using custom code written in python v3.9.12, using the following packages: pytorch v2, numpy v 1.19.2, scipy v1.6.2, scikit-learn v1.0.2. Behavioural experiment stimuli were created using custom code written in JavaScript. Images of 3D object stimuli were rendered using Blender 2.79. Point clouds were sampled from these stimuli using CloudCompare 2.12. Human behavioral data, model results, and original stimulus materials can be downloaded from: https://github.com/taylorwwebb/visiPAM.
Data analysis	All statistical analyses were carried out using custom code written in python v3.9.12. Analysis code is available at: https://github.com/taylorwwebb/visiPAM. Model code is not accessible to the public due to the requirements of the funding agency (AFRL). However, it will be made available to individual members of the research community upon request. Researchers can request code by contacting Hongjing Lu.

For manuscripts utilizing custom algorithms or software that are central to the research but not yet described in published literature, software must be made available to editors and reviewers. We strongly encourage code deposition in a community repository (e.g. GitHub). See the Nature Portfolio guidelines for submitting code & software for further information.

Data

Policy information about availability of data

All manuscripts must include a data availability statement. This statement should provide the following information, where applicable:

- Accession codes, unique identifiers, or web links for publicly available datasets
- A description of any restrictions on data availability
- For clinical datasets or third party data, please ensure that the statement adheres to our policy

The Pascal Part Matching (PPM) dataset is available for download at: https://allenai.github.io/one-shot-part-labeling/ppm/. Human behavioral data, model results, and original stimulus materials can be downloaded from: https://github.com/taylorwwebb/visiPAM.

Research involving human participants, their data, or biological material

Policy information about studies with human participants or human data. See also policy information about sex, gender (identity/presentation), and sexual orientation and race, ethnicity and racism.

Reporting on sex and gender	The gender of the participants in our behavioral study is reported as follows: 'Fifty-nine participants (mean age = 20.55 years; 51 female) were recruited...'
Reporting on race, ethnicity, or other socially relevant groupings	No data concerning race or ethnicity are reported.
Population characteristics	See above.
Recruitment	Participants were recruited as part of a subject pool for undergraduate psychology courses. Students in undergraduate psychology courses at UCLA are required to either participate in psychology experiments, or to write a report summarizing psychology research. They are informed of this requirement upon enrolling in the course. Most students choose to fulfill this requirement by participating in studies. There are not any significant sources of self-selection bias as a result of this recruitment procedure.
Ethics oversight	All experiments were approved by the UCLA Institutional Review Board, and all participants provided informed consent.

Note that full information on the approval of the study protocol must also be provided in the manuscript.

Field-specific reporting

Please select the one below that is the best fit for your research. If you are not sure, read the appropriate sections before making your selection.

☐ Life sciences ☒ Behavioural & social sciences ☐ Ecological, evolutionary & environmental sciences

For a reference copy of the document with all sections, see nature.com/documents/nr-reporting-summary-flat.pdf

Behavioural & social sciences study design

All studies must disclose on these points even when the disclosure is negative.

Study description	This study investigates visual analogical reasoning using quantitative behavioral measures from human participants.
Research sample	Fifty-nine participants (mean age = 20.55 years; 51 female) were recruited from the Psychology Department subject pool at the University of California, Los Angeles. This sample is representative of undergraduate students in the United States. This sample was chosen because it was not feasible to include other groups in the study, and because the basic cognitive processes under investigation (analogical reasoning) did not require recruitment of a specific population. Please note that although the study used a pre-existing dataset for evaluating the computer model (the Pascal Part Matching dataset), this dataset does not include human behavioral data. All human behavioral data was collected specifically for the purposes of this study.
Sampling strategy	All experimental manipulations were at the within-subject level, therefore no sampling procedure was needed (all participants provided responses in all conditions). No statistical methods were used to pre-determine the sample size. We recruited as many participants as was feasible given the time frame of the experiment.
Data collection	Data was collected in an online experiment. Participants performed the experiment on their own computers. The researchers were not blind to the study's hypotheses, but they did not interact with participants, as the study was performed online. Only the participant was present when performing the study (neither the researchers nor anyone else was present).
Timing	Data was collected between October 2021 and January 2022.
Data exclusions	Five out of the 59 participants were removed from analysis either because they indicated they were not serious,

Data exclusions	or because they moved fewer than 30% of the markers in the entire experiment. Thirteen additional participants were removed because they did not move any of the markers in at least one of the conditions. Thus, data from a total of 41 participants were included in the analyses.
Non-participation	No participants dropped out or declined to participate.
Randomization	Participants were not allocated into experimental groups. All experimental manipulations were at the within-subject level.

Reporting for specific materials, systems and methods

We require information from authors about some types of materials, experimental systems and methods used in many studies. Here, indicate whether each material, system or method listed is relevant to your study. If you are not sure if a list item applies to your research, read the appropriate section before selecting a response.

Materials & experimental systems

n/a	Involved in the study
☒	☐ Antibodies
☒	☐ Eukaryotic cell lines
☒	☐ Palaeontology and archaeology
☒	☐ Animals and other organisms
☒	☐ Clinical data
☒	☐ Dual use research of concern
☒	☐ Plants

Methods

n/a	Involved in the study
☒	☐ ChIP-seq
☒	☐ Flow cytometry
☒	☐ MRI-based neuroimaging
